# Ligation-assisted endoscopic full-thickness resection combined with presuture for resection of a colonic gastrointestinal stromal tumor

**DOI:** 10.1055/a-2501-3181

**Published:** 2025-01-14

**Authors:** Zhaohui Liu, Hualin Li, Yue Chao, Dayong Sun, Ruinuan Wu

**Affiliations:** 1 Department of Gastroenterology, Shenzhen Second People’s Hospital, First Affiliated Hospital of Shenzhen University Health Science Center, Shenzhen, China; 2 Department of Pathology, Shenzhen Second People’s Hospital, First Affiliated Hospital of Shenzhen University Health Science Center, Shenzhen, China

Ligation-assisted endoscopic full-thickness resection (L-EFTR) is commonly used to remove tumors of the gastric muscularis propria 1 2 but rarely to remove tumors of the colonic muscularis propria because of the limited visual field after colon perforation and the high incidence of peritonitis. Here, we report a novel method, named “ligation-assisted endoscopic full-thickness resection combined with presuture” (L-EFTR-P) ( Video 1 ), for the endoscopic full-thickness resection of a gastrointestinal stromal tumor (GIST) in the transverse colon. With the use of L-EFTR-P, closure of the colonic perforation was quick and easy.

Ligation-assisted endoscopic full-thickness resection combined with presuture resection for a gastrointestinal stromal tumor in the transverse colon of a 61-year-old woman.Video 1

A 61-year-old woman underwent a screening colonoscopy, during which a 6-mm submucosal tumor was discovered in the transverse colon ( Fig. 1 **a** ). Endoscopic ultrasound examination revealed that the tumor originated from the muscularis propria ( Fig. 1 **b** ).

**Fig. 1:**
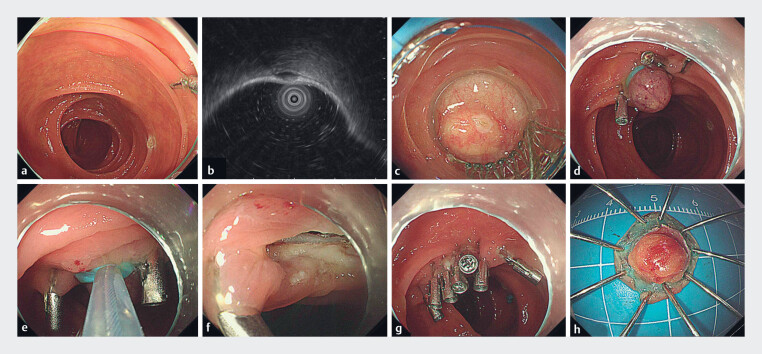
Ligation-assisted endoscopic full-thickness resection combined with presuture (L-EFTR-P) for a gastrointestinal stromal tumor in the transverse colon. **a** A 6-mm submucosal tumor was detected in the transverse colon. **b** Endoscopic ultrasound examination revealed that the tumor originated from the muscularis propria. **c** The ligation device was attached to the front end of the endoscope, and the tumor was surrounded completely by the cap. **d** The tissue clip was clamped close to the rubber band. **e** The snare was tightened under the rubber band. **f** A linear perforation was observed. **g** The perforation was sutured with tissue clips. **h** The excised specimen was removed, and the size was measured.

The L-EFTR-P procedure was performed as follows. The ligation device (M00542251; Boston Scientific, Marlborough, Massachusetts, USA) was attached to the front end of the endoscope, and the tumor was surrounded completely by the cap ( Fig. 1 **c** ). After continuous suction, the entire tumor was positioned within the cap, the rubber band was subsequently released for ligation, and the tissue clips (POCC-D-26-195; Micro-Tech (Nanjing), Nanjing, China) were clamped close to the rubber band ( Fig. 1 **d** ). A snare (M00561231; Boston Scientific) was tightened under the rubber band ( Fig. 1 **e** ). The tumor was removed, and a linear perforation was observed ( Fig. 1 **f** ). The perforation was sutured with tissue clips ( Fig. 1 **g** ). The excised specimen was removed, and the size was measured ( Fig. 1 **h** ). The patient had no postoperative complications, including abdominal pain, chills, fever, or intra-abdominal bleeding. Postoperative pathology confirmed that the tumor was a GIST.

The L-EFTR-P technique can effectively partially suture the perforation before tumor resection and thus avoid the occurrence of large perforations, representing a safe, effective, and feasible endoscopic method for tumor resection in the colonic muscularis propria.

Endoscopy_UCTN_Code_TTT_1AQ_2AD_3AF

## Notes

Endoscopy E-Videos https://eref.thieme.de/e-videos
*E-Videos* is an open access online section of the journal *Endoscopy* , reporting on interesting cases and new techniques in gastroenterological endoscopy. All papers include a high-quality video and are published with a Creative Commons CC-BY license. Endoscopy E-Videos qualify for HINARI discounts and waivers and eligibility is automatically checked during the submission process. We grant 100% waivers to articles whose corresponding authors are based in Group A countries and 50% waivers to those who are based in Group B countries as classified by Research4Life (see: https://www.research4life.org/access/eligibility/ ). This section has its own submission website at https://mc.manuscriptcentral.com/e-videos .
